# Arthralgia and Osteolytic Lesions Associated with Traumatic Pancreatitis in a 10-Year-Old Girl

**DOI:** 10.1155/2009/950687

**Published:** 2009-03-11

**Authors:** Masayuki Obatake, Yusuke Yamane, Takayuki Tokunaga, Yasuaki Taura, Yukio Inamura, Takeshi Nagayasu

**Affiliations:** Division of Surgical Oncology, Nagasaki University Graduate School of Biomedical Sciences, 1-7-1 Sakamoto, Nagasaki 852 8501, Japan

## Abstract

A case of traumatic pancreatitis with subsequent joint pain and osteolytic lesions is presented. A 10-year-old girl was admitted to our hospital with abdominal pain caused by blunt epigastric injury. She was diagnosed with traumatic pancreatitis, and multiple pancreatic pseudocysts subsequently developed. Two weeks after admission, she complained of joint pain, and MR revealed osteolytic lesions of both knee joints. On the 58th day, endoscopic transgastric pseudocyst drainage was performed. Joint pain and osteolytic lesions resolved rapidly, in parallel with the decrease in serum amylase level and pseudocyst size.

## 1. Introduction

Abdominal pain or pseudocyst formation is relatively frequent after traumatic pancreatic injury in children, but complications of arthritis and osteolytic lesions are uncommon in this situation [1–3]. We report a 10-year-old patient with acute traumatic pancreatitis who developed joint pain and osteolytic lesions, which resolved after endoscopic transgastric pseudocyst drainage.

## 2. Case Report

**Figure 1:**
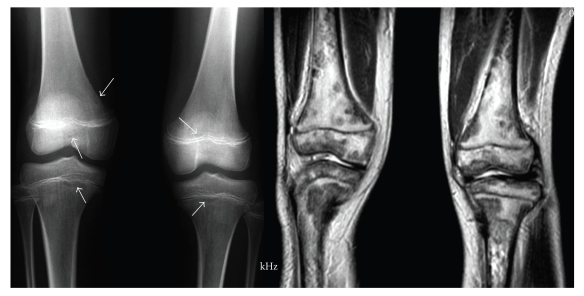
AP and MR image of both knees. AP film of both knees shows osteolytic lesions as well-marginated lucencies in femurs and tibias. Sagittal T2-weighted MR image of both knees shows multiple nodular low-density lesions.

A 10-year-old girl was referred to our emergency department because of increasing abdominal pain. A couple of hours prior to admission, she had slipped while walking along a steel fence, thereby sustaining a blunt epigastric injury. She had moderate abdominal distension and tenderness. Laboratory examination revealed a peripheral white blood cell (WBC) count of 14 000/mm^3^ and serum amylase of 296 IU/L. A tentative diagnosis of traumatic pancreatitis was confirmed by abdominal computed tomography (CT) showing a hematoma anterior to the pancreas and peripancreatic effusion. Pancreatic pseudocyst formation was not demonstrated on initial ultrasonography. She was admitted and managed conservatively with nasogastric decompression and bowel rest. Gabexate mesilate and antibiotics were started, and on the 6th hospital day, intravenous hyperalimentation was commenced. Two weeks after admission, she complained of pain in both knee joints, and serum amylase was found to be elevated at 6024 IU/L. Radiography demonstrated osteolytic changes in both distal femurs. In addition, magnetic resonance (MR) imaging of both knee joints demonstrated multiple nodular low-density lesions on T1- and T2-weighted images in both distal femurs and both proximal tibias and fibulas (Figure 1). On the 40th hospital day, incapacitating abdominal pain developed with elevation of serum amylase to 8140 IU/L. On the 51st hospital day, CT and abdominal ultrasonography revealed multiple pseudocysts (diameters ranging from 2 cm to 8 cm) bulging into the posterior gastric wall. Communication among the cysts was seen in the body of the pancreas. The biliary tract was not dilated. MR cholangiopancreatography showed neither rupture nor stenosis of the major pancreatic duct. Despite improvement of the initial abdominal pain, she complained of persisting pain in both knee joints. On the 58th hospital day, she underwent endoscopic transgastric pseudocysts drainage with a 7-Fr stent using endoscopic ultrasonography. The stent had flaps at both ends to prevent dislocation. She had an uneventful postoperative course and started eating on the third postoperative day. After this drainage, serum amylase normalized within one week. Repeat CT and abdominal ultrasonography revealed rapid shrinkage of the pancreatic pseudocysts. The pain in both knee joints resolved in 2 weeks, but radiographic examination and MR images demonstrated the persistence of osteolytic lesions around both knee joints. She remained in the hospital for another 48 days following the pseudocyst drainage and had neither pain nor difficulty in walking at the time of discharge. The internal drainage tube was passed unnoticed before the 3-month postoperative check-up.

## 3. Discussion

Bone marrow involvement in acute pancreatitis was first described by Ponfick in 1872 [4]. Since then, the presence of bone lesions associated with pancreatitis in adults has been reported, but it is rare in children. In 1972, Keating et al. reported osteolytic lesions complicating acute pancreatitis in a case of child abuse [5].

The mechanism for the formation of osteolytic lesions in this situation has not been clarified. Osteolytic changes seen in pancreatitis are thought to result from peripheral ischemic necrosis caused by intravascular thrombosis due to direct injury to the blood vessels by pancreatic enzymes [6–8]. It has also been speculated that fat cells located in bone marrow, periosteum, and around joints are directly damaged by pancreatic enzymes and embolize into the vessels [4, 8]. Acute pancreatitis in children can be classified by etiology into four major groups: traumatic, systemic disease related, drug-induced, and idiopathic acute pancreatitis. A number of reported cases with osteolytic changes in pancreatitis were relevant to the traumatic pancreatitis. It is conceivable that osteolytic changes seen in pancreatitis occur because of massive pancreatic enzyme influx into the systemic circulation caused by the pancreatic damage by trauma. Osteolytic changes are rare in patients with nontraumatic pancreatitis.

Osteolytic changes are usually found at least 3 to 4 weeks after the initial episode and are accompanied by joint pain [5, 7, 9]. The osteolytic lesions occur symmetrically at the metaphyses of long bones such as the femur, tibia, radius, and ulna and gradually resolve as serum amylase level falls [5, 10]. Radiography demonstrates multiple mottled defects, and technetium pyrophosphate scintigraphy reveals increased uptake in pathological areas. MR images demonstrate fat necrosis in the bone marrow as decreased signal intensity on both T1- and T2-weighted images [11, 12]. The radiological changes remain for several months after the improvement of clinical symptoms. In our case, the bone lesions developed 2 weeks following the diagnosis of traumatic pancreatitis.

Differential diagnosis of these osteolytic changes in children includes osteomyelitis, trauma, tumor metastasis, and child abuse. Diagnosis of osteomyelitis is excluded by multiple bone lesions and negative blood cultures. Traumatic osteolytic changes should be ruled out from the history and by normal radiographic appearance of the bones on admission. The possibility of child abuse must be entertained even when there is no history of trauma. Osteolytic changes in pancreatitis occur 3 to 4 weeks after the initial insults to the pancreas. This delay may obscure the primary etiology of pancreatitis because most symptoms of pancreatitis have usually abated by this stage. Specific treatment is not necessary for these unusual osteolytic changes. Improvement of the pancreatitis is followed by complete resolution of bone lesions and arthritis in several months to a year, without sequelae [1, 5].

In conclusion, although osteolytic changes are a rare complication of traumatic pancreatitis, clinicians should be aware of this possibility.
